# Quantification of marine benthic communities with metabarcoding

**DOI:** 10.1111/1755-0998.13536

**Published:** 2021-11-01

**Authors:** Lise Klunder, Judith D. L. van Bleijswijk, Loran Kleine Schaars, Henk W. van der Veer, Pieternella C. Luttikhuizen, Allert I. Bijleveld

**Affiliations:** ^1^ Department of Coastal Systems NIOZ Royal Netherlands Institute for Sea Research AB Den Burg Texel The Netherlands; ^2^ Marine Evolution and Conservation Groningen Institute of Life Sciences University of Groningen CC Groningen The Netherlands; ^3^ Department of Marine Microbiology and Biogeochemistry NIOZ Royal Netherlands Institute for Sea Research AB Den Burg Texel The Netherlands

**Keywords:** abundance, biomass, eDNA, metabarcoding, next‐generation sequencing, quantification

## Abstract

DNA metabarcoding methods have been implemented in studies aimed at detecting and quantifying marine benthic biodiversity. In such surveys, universal barcodes are amplified and sequenced from environmental DNA. To quantify biodiversity with DNA metabarcoding, a relation between the number of DNA sequences of a species and its biomass and/or the abundance is required. However, this relationship is complicated by many factors, and it is often unknown. In this study, we validate estimates of biomass and abundance from molecular approaches with those from the traditional morphological approach. Abundance and biomass were quantified from 126 samples of benthic intertidal mudflat using traditional morphological approaches and compared with frequency of occurrence and relative read abundance estimates from a molecular approach. A relationship between biomass and relative read abundance was found for two widely dispersed annelid taxa (*Pygospio* and *Scoloplos*). None of the other taxons, however, showed such a relationship. We discuss how quantification of abundance and biomass using molecular approaches are hampered by the ecology of DNA i.e. all the processes that determine the amount of DNA in the environment, including the ecology of the benthic species as well as the compositional nature of sequencing data.

## 1 INTRODUCTION

Marine benthic ecosystems are characterized by high biodiversity and are of global importance to climate, nutrient cycling and primary and secondary productivity (Austen et al., 2002; Covich et al., 2004; Snelgrove, 1997). From the intertidal to the deep sea, benthic fauna are central to the maintenance of ecosystem services, whereby a high diversity is thought to maintain a positive interaction among species and promoting stability and resistance to ecosystem functioning (Danovaro et al., 2008; Leduc and Pilditch, 2013; Levin et al., 2001).

Effective monitoring of the benthic fauna is a first crucial step towards conservation of the marine benthic ecosystems (Patrício et al., 2009). Traditionally, benthic biodiversity assessments are based on the morphological identification of species (see for instance Compton et al., 2013; Diaz et al., 2004). These morphological inventories are time‐consuming, require taxonomic expertise which is scarce and are often limited to macrofauna species (Beukema & Dekker, 2020; Bucklin et al., 2011; Cardoso et al., 2011; Cowart et al., 2015). Thus, there is a need for methods that can assess marine benthic biodiversity in a rapid and cost‐effective yet detailed and accurate manner (Aylagas et al., 2018).

In recent years, DNA metabarcoding methods have been successfully implemented in various studies to assess marine benthic biodiversity. For instance, Chariton et al. (2015) used this approach to assess the ecological condition of estuaries, whereas Lanzén et al. (2016) was able to identify effects of offshore oil‐drilling activities using eukaryotic metabarcoding. DNA metabarcoding provides the opportunity to assess the benthic community in a replicable manner that allows for the simultaneous recovery of a wide variety of taxa from all size classes without first isolating any organisms, facilitating rapid biodiversity monitoring (Taberlet, Coissac, et al., 2012). DNA metabarcoding most often relies on the extraction of DNA from a matrix of choice ‐ sediment, water, air or a mixture of organisms ‐ followed by the amplification of a DNA barcode via PCR (Hebert et al., 2003; Taberlet, Coissac, et al., 2012). These DNA barcodes are sequenced and taxonomically assigned against globally available databases to infer information about the community (Pruesse et al., 2007).

Morphological methods to identify benthic macrofauna rely on the sorting and identification of individual specimens. These morphological methods most often produce quantitative data including measurements on heterogeneity diversity, for example, the proportional abundances of species (Gray, 2000). The ability to acquire such quantitative data, as opposed to qualitative data only, can greatly enhance the power of ecological studies as it provides more insights on the biodiversity and/or the conservation status of a species (Gray, 2000; Mace et al., 2008). Comparable to morphological studies, results in metabarcoding studies are frequently reported in (semi‐)quantitative terms. In these studies, it was assumed these quantitative measurements would relate to the quantifications used in traditional benthic surveys (Porazinska et al., 2010). Two main approaches can be distinguished in quantifying communities using a molecular method: a frequency of occurrence approach and a relative read abundance approach. The frequency of occurrence approach counts the presence of a taxon over multiple samples and assumes that a higher occurrence reflects a higher abundance of this taxon in the environment (Deagle et al., 2019). The frequency of occurrence approach has been used extensively in dietary studies (e.g., Berry et al., 2017; De Barba et al., 2014) but also in biodiversity studies (e.g., Chariton et al., 2015; Jeunen et al., 2019). The relative read abundance approach uses percentages of the total number of reads as an estimate of biomass (Lamb et al., 2019) and has been widely adopted in marine benthic studies (e.g., Cahill et al., 2018; Sinniger et al., 2016). A meta‐analysis on 22 metabarcoding studies by Lamb et al. (2019) showed a weak but positive relationship between relative read abundance and biomass and such a relationship was also reported for studies on invertebrate species (Elbrecht et al., 2017; Porazinska et al., 2010).

Nonetheless, using eDNA metabarcoding studies in a quantitative manner is debatable. The quantification of macrofauna species from small environmental samples, normally used in metabarcoding studies, is challenging due to several factors. Firstly, there a methodological issues in metabarcoding studies that include the effectiveness of different DNA extraction approaches on different communities (Brannock & Halanych, 2015; Klunder et al., 2019); primer biases (Deagle et al., 2014; Piñol et al., 2015; Piñol et al., 2019) and biases induced in bioinformatic pipelines (Nichols et al., 2018; Plummer & Twin, 2015; Richardson et al., 2017). Secondly, ecological and biological issues can arise. Quantitative measurements are derived from the presence of environmental DNA (eDNA), where DNA fragments are used as a proxy for the presence of a specimen (Harrison et al., 2019). This presence of eDNA is affected by, for example, the shedding rates of DNA by the source organism. Specifically, shedding rates depend on morphological and physiological characteristics or seasonal patterns and can increase up to 100‐fold at certain times (Barnes & Turner, 2016; Harrison et al., 2019). Furthermore, the variability of DNA per gram tissue varies due to variable tissue cell density (Pompanon et al., 2012) or the transport of eDNA through the environment (Kelly et al., 2018). Lastly, there is no true independence between the quantitative measurements of species within an eDNA data set due to the compositional base of sequencing data. This compositional base is due to the maximum limit of reads which can be translated during sequencing,and leads to a negative correlation bias between species abundancies (Gloor et al., 2017). The frequency of occurrence approach is only based on detections and not on the compositional data set, and, this is hypothetically not under influence of the negative correlation bias.

To summarize, metabarcoding studies can aid biodiversity assessments. However, the quantitative abilities of such studies are unknown and questionable. The aim of this study is to explore to what extent traditional and metabarcoding approaches align in assessing abudances and biomass of marine macrofauna communities. Specifically, whether metabarcoding outputs from sediment samples can be used as a quantitative estimate for abundance and biomass of benthic macrofauna in the intertidal Dutch Wadden Sea. For this study, we chose to use a fast and easy sampling approach for the collection of eDNA samples in which environmental DNA is extracted from small sediment cores (Klunder et al., 2019); an approach which can readily be adopted in future monitoring programs. Simultaneously, we collected morphological‐based data to estimate species occurrence and abundance. The first aim of the study was to examine whether detection rates for benthic macrofauna species were comparable between molecular and traditional analyses. Following that, we compared the morphological approach with the DNA metabarcoding approach to test the reliability of both a frequency of occurrence approach and relative read abundance approach for estimating abundances and biomass.

## 2 MATERIALS AND METHODS

### 2.1 Sampling

**FIGURE 1:**
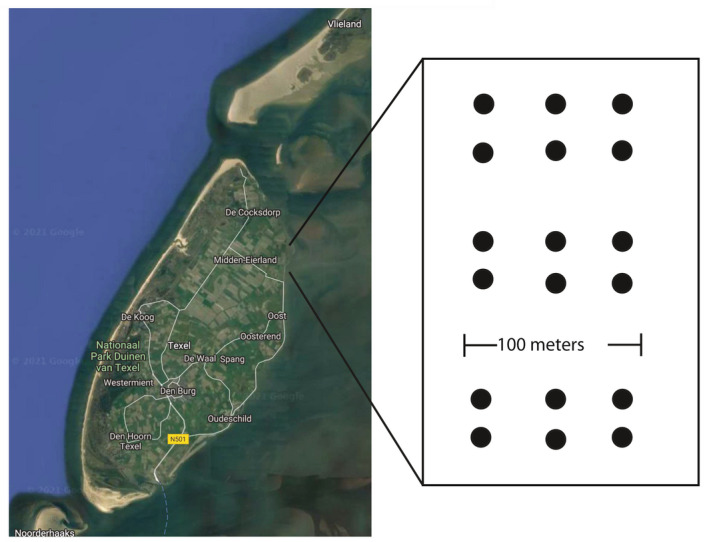
Map of Texel, showing the sampling locations at the intertidal mudflats, NE of Texel. Also, a graphical display of the sampling scheme is shown. All points were sampled in 2016 at: 6 June and 14 November and in 2017: 13 March, 9 May, 23 May, 6 June and 26 June

Divided over seven sampling events between June 2016 and July 2017, a total of 126 locations were sampled on tidal flats north‐east of the isle of Texel in the western part of the Dutch Wadden Sea (N53°06' E4°54'). Each sampling event comprised 18 stations within a 500 m spatial range (Figure 1). At each station, two cores were taken; one larger core for the morphological identification of macrofauna (177 cm^2^, 25–30 cm depth) and one smaller for the molecular identification (5.6 cm^2^, 10 cm depth). The two cores were sampled directly adjacent to each other and were taken simultaneously.

### 2.2 Morphological approach

The samples for morphological analyses were washed over a 1 mm mesh sieve in the field and stored in the freezer at –20°C. In the laboratory, samples were thawed and preserved in a 4% formaldehyde solution with Bengal rose (~2.5 mg/L). Species were sorted by hand and identified. Taxonomic identification was based on the NIOZ reference collection of local benthic macrofaunal species conform WoRMS Editorial Board (2019). Individuals were counted and biomass (g) was determined as ash‐free dry mass of the flesh following Compton et al. (2013). To minimise weighing error for small biomasses, specimens of small taxa such as *Tharyx* sp., *Eteone* sp., Oligochaeta and *Pygospio* sp. where only weighted for a minimum of four individuals and *Heteromastus* sp. was only weighted with a minimum of two individuals and average biomass estimates from the data set were used instead.

### 2.3 Molecular approach

To remove extracellular DNA, the entire samples for molecular identification were rinsed twice with a saturated phosphate‐buffer (Na_2_HPO_4_; 0.12 M; pH ≈ 8). During this process, the sediments were submerged in the buffer for 5 min to release the extracellular DNA from the sediments (Taberlet, Prud'homme, et al., 2012) and the supernatant was removed subsequently. The extracellular DNA bound to the sediment by phosphate bridges is less susceptible to degradation and might lead to an overpresentation of the actual living community due to a temporal buffering (Corinaldesi et al., 2008; Guardiola et al., 2016). The sediments were then cryodesiccated and ground in liquid nitrogen.

Ten grams of the homogenized sediment served as starting material for the DNA extraction. DNA was extracted using the Powermax Soil DNA isolation kit (Qiagen Inc.) following the manufacturer's instructions. DNA from all extractions, as well as four identical mock samples (Table S1) were used as template for amplification in triplicate. A 450 base pair (bp) part of the nuclear small ribosomal subunit (18S) was amplified using the oligonucleotides F04 and R22mod as primer pair (Sinniger et al., 2016). This primer pair was chosen from a set of six primers tested, both 18S and cytochrome c oxidase subunit I (COI). The primers were found to amplify all taxa after an in vitro test on a set of macrofauna species from the experimental area (Tables S2 and S3). All forward and reverse primers were extended with 12nt unique barcodes based on the NEXTflex‐HT barcodes as to prevent false reads and/or sample assignments due to chimera's and taq‐jumps. The 18S gene was amplified in a 50 μl volume reaction, containing 0.6 μM of each primer, 0.2 μM dNTP, 800 ng/μl BSA, 1 U Phusion high‐fidelity DNA polymerase (Thermo Scientific Inc.), 1× Phusion HF buffer (Thermo Scientific Inc.) and 5 μl of DNA extract. The thermal cycle conditions were as follows: an initial cycle of 30 s at 98°C; followed by 27 cycles, each comprised of 10 s at 98°C, 20 s at 60°C and 30 s at 72°C, followed by a single cycle of 5 min at 72°C. The PCR products as well as four blank PCR controls were excised from a 1% agarose gel, purified using the Qiaquick Gel Extraction Kit (Qiagen, Inc.) and quantified with a Qubit 3.0 fluorometer (Qiagen Inc.). All samples were pooled in equimolar quantities. The pooled sample was then subjected to a final purification using MinElute PCR Purification columns (Qiagen Inc.) as described by the manufacturer. The pooled sample was sequenced at Useq on an Illumina MiSeq using the 2× 300 bp V3 kit.

### 2.4 Bioinformatics

Raw sequences with a quality score ≤30 over 75% of the nucleotide positions were discarded using the fastq_quality_filter script in the FASTX‐Toolkit (https://hannonlab.cshl.edu/). Quality filtered reads were demultiplexed using the split_libraries.py script in QIIME (Caporaso et al., 2010), allowing zero mismatches in both the forward and reverse barcode label. Subsequently, reads were checked for chimera's and dereplicated in VSEARCH (Rognes et al., 2016) and unique sequences were discarded. The remaining sequences were clustered with a 98% similarity cutoff. This cutoff was found sufficient for genus‐level identification for the macrofauna species in our reference library for this area. Singletons were discarded and the remaining out clusters were taxonomically assigned using the RDP Classifier (Wang et al., 2007) with a minimum confidence of 0.8 against the SILVA 18S rRNA database (release 132, Pruesse et al., 2007) and our local reference database (Table S3 and Genbank accession numbers MZ709983–MZ710042). Our local reference database covers all macrofauna species found with the morphological approach. Only reads from taxonomic families containing macrofauna species were retained (see Table 1), reads from other families and reads assigned at a higher taxonomic level were omitted. OTUs assigned at the family level were compared against the NCBI database (https://blast.ncbi.nlm.nih.gov, accessed at 10/2019) using blastn; the OTUs which returned a match with a percent identity >99% at the genus level were assigned accordingly. Raw Illumina sequences were deposited in the European Nucleotide Archive (ENA accession number: PRJEB46793).

**TABLE 1:** Frequency of detections per genus among a total of 126 samples for morphological (Morpho) and molecular (DNA) methods

Taxonomy			Detections	
Phylum	Family	Genus	Morpho	DNA
Annelida	Nereididae	Alitta	1	3
	Arenicolidae	Arenicola	104	104
	Capitellidae	Capitella	104	107
	Echiuridae	Echiurus	‐	8
	Phyllodocidae	Eteone	68	20
	Phyllodocidae	Eumida	‐	6
	Syllidae	Exogone	‐	6
	Glyceridae	Glycera	‐	10
	Nereididae	Hediste	9	46
	Capitellida	Heteromastus	42	98
	Terebellidae	Lanice	2	89
	Magelonidae	Magelona	3	1
	Spionidae	Marenzelleria	40	10
	Nephtyidae	Nephtys	6	3
	Oligochaeta sp.	Oligochaeta	58	101
	Phyllodocidae	Phyllodoce	4	2
	Spionidae	Polydora	‐	3
	Spionidae	Pygospio	76	94
	Orbiniidae	Scoloplos	122	110
	Spionidae	Spio	‐	12
	Spionidae	Streblospio	‐	20
	Cirratulidae	Tharyx	46	123
Arthropoda	Carcinidae	Carcinus	2	2
	Crangonidae	Crangon	38	3
	Gammaridae	Gammarus	3	‐
	Urothoidae	Urothoe	81	4
Mollusca	Tellinidae	Macomangulus	‐	3
	Cardiidae	Cerastoderma	‐	18
	Ostreidae	Magallana	‐	36
	Pharidae	Ensis	2	80
	Tellinidae	Limecola	16	5
	Myidae	Mya	1	35
	Mytilidae	Mytilus	‐	54
	Hydrobiidae	Peringia	2	29
	Veneridae	Petricolaria	‐	13

### 2.5 Data analysis

All statistical analyses and data visualisations were performed in R 3.5.2. For the morphological approach, count and biomass data were calculated at the genus level, resulting in a total number of individuals and a total biomass per taxonomic genus per sample. Both the abundance and biomass data showed a skewed distribution and hence were square root transformed. To prevent a bias in the molecular data set between samples due to variable sequencing depths per sample, read numbers per genus for all molecular samples were transformed into a relative abundance of reads per sample. Although read numbers per samples differed, no relation was found between sequencing depth and species richness (*r*_(124)_ = –.36, *p* = .72) Therefore relative abundancies were chosen over rarefaction to conserverare OTUs (McMurdie & Holmes, 2014) whereas relative read abundance transformations preserve all read calls (Lanzén et al., 2016). For each taxon, their presence per sample was scored and the occurrence was calculated as the sum of total detections divided by the total number of samples. Unfortunately, detection rates for all taxa within the arthropod and mollusc phyla were too low (detection rate < 20% for both approaches) and highly biased in either the morphological or the molecular methods to test these assumptions within these taxa. Therefore, the reliability of the quantitative approaches could only be tested on annelid taxa.

To test the reliability of the frequency of occurrence approach, the assumption that a higher occurrence in the morphological method corresponds to a higher detection probability in the molecular method was tested. The square root of the abundance data as derived from the morphological method was compared to the occurrence in the molecular method based on a logistic regression using the popbio package in R. The strength of the logistic regression was assessed using Wald's‐χ^2^ and a receiver operating characteristics (ROC) curve. The ROC curve shows the sensitivity (true positive rate) of the logistic regression as a function of the nonspecificity (false positive rate) and was built using R package ROCR. The reliability of the relative read abundance approach was tested using a linear regression. This linear regression was based on the square root of the biomass data for the morphological method and the relative read abundance data for the molecular method.

## 3 RESULTS

### 3.1 Detection probability

**FIGURE 2:**
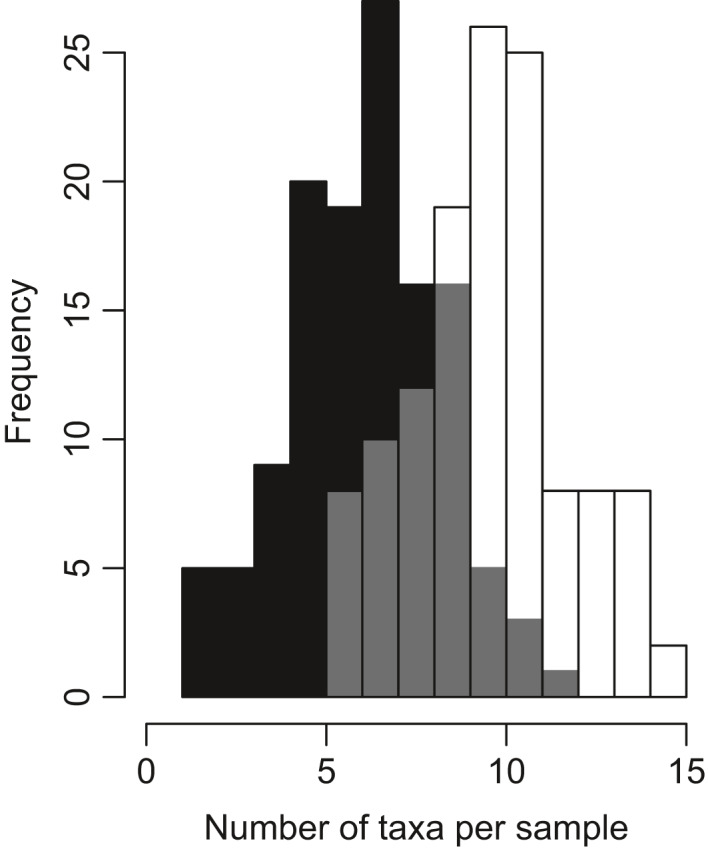
Histogram showing frequency distribution of number of macrofauna taxa (genus) identified per sample for morphological (black) and molecular method (white). The grey area indicates overlap

In total, 23 and 35 macrofauna taxa (genus‐level) could be identified from the morphological and molecular samples, respectively (Table 1) of which 22 taxa were identified by both approaches. The molecular approach detected seven extra taxa attributed to Annelida and five extra Mollusca taxa compared to the morphological approach, whereas the arthropod *Gammarus* was only identified by the morphological approach but not with the molecular approach. The annelid taxa exclusively detected by the molecular approach mostly include smaller specimens such as *Echiura*, *Eumida*, *Spio*, *Streblospio and Polydora*. Moreover, the genus *Polydora* is known to include parasitic species and might therefore have been present concealed in its host species. Figure 2 shows that the average number of taxa detected per sample was significantly higher (paired *t* test, *t*_125_ = 13.1, *p* < .001) for the molecular approach (mean = 10.0 ± 2.2) than for the morphological approach (mean = 6.6 ± 2.1).

**FIGURE 3:**
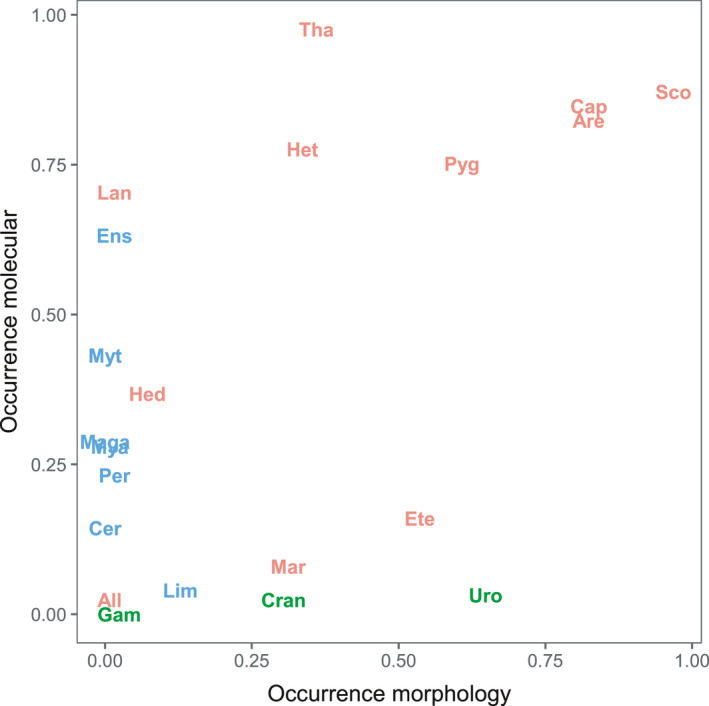
Occurrence per taxon, calculated as the sum of detections divided by the total number of samples (*n* = 126) for both the morphological method (x‐axis) and the molecular method (y‐axis). Taxa detected belonged to three phyla: Annelida (red), Arthropoda (green) or Mollusca (blue)

The detection rates (calculated as sum of detections divided by the number of samples) for the 21 most abundant species are visualized in Figure 3. A clear discrepancy could be seen for the mollusc and arthropod taxa. The detection rates for arthropod taxa were higher in the morphological samples. For instance, *Urothoe* was detected in 64% of the samples for the morphological approach and only in 3% of the samples in the molecular approach. Mollusc species were overall detected at higher rates in the molecular samples. Five mollusc taxa which were detected in the molecular samples (i.e., *Macomangulus* [2.4%], *Cerastoderma* [14.3%], *Magallana* [28.6%], *Littorina* [0.79%] and *Petricolaria* [10.3%]) were never detected in the morphological samples. Detection rates for the annelids were higher for both approaches compared to the detection rates of the other phyla. Except for *Eteone* and *Marenzelleria*, detection rates for the annelid taxa were higher in the molecular approach compared to the detection rates in the morphological approach.

### 3.2 Frequency of occurrence and relative read abundance

**FIGURE 4:**
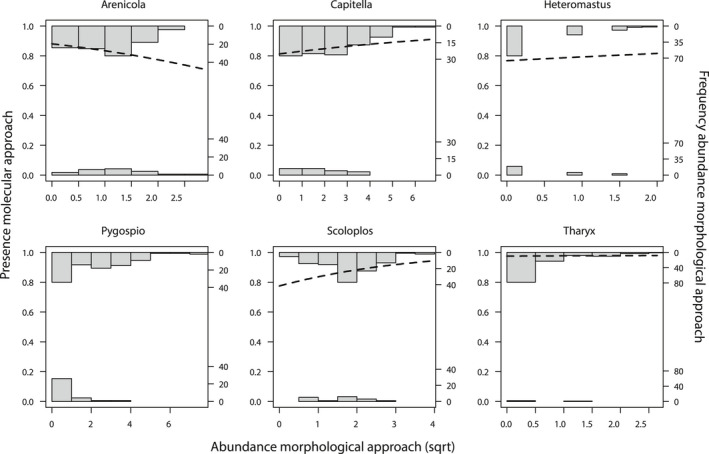
Logistic regression and abundance distribution histograms for six annelid taxa. The histograms on the top show the abundance distribution if the species was also found in the molecular samples, the histograms on the bottom is the species was not present in the molecular data set, the frequency is shown on the y‐axis. The abundance distribution as derived from the morphological method are shown in histograms at the sqrt‐transformed x‐axis

To test the reliability of the frequency of occurrence approach, the abundance data as derived from the morphological method were modelled using a logistic regression against the occurrence in the molecular data set. Only the abundance data for *Pygospio*, as derived from the morphological approach, was a significant predictor for the detection of this taxon within the molecular method (Figure 4, Table 2). For *Pygospio*, per unit of increase in abundance, the odds of a detection in the molecular approach increased by a factor 1.34 (Wald‐*χ* ^2^_(124)_ = 3.27, *p* = .001). Most of the other annelid taxa did, however, show a positive relationship between the abundance in the morphological approach and occurrence in the molecular inventory. The only exception was *Arenicola* for which the odd‐ratio of occurrence in the molecular approach declined with increasing abundance.

**TABLE 2:** Summary of logistic regression and linear regression as used for frequency of occurrence and relative read abundance approaches compared to the traditional morphological methods, respectively

Taxa	Frequency of occurrence approach						Relative read abundance approach		
	Odd‐ratio	2.5%	97.5%	Wald‐χ^2^	*p*‐value	AUC (%)	Slope	*R* ^2^ (%)	*p*‐value
Arenicola	–0.84	–0.62	–1.14	1.37	.242	55	–0.35	11	.030
Capitella	1.05	0.98	1.16	1.25	.263	55	0.68	1	.630
Heteromastus	1.19	0.73	2.13	0.42	.518	52	0.59	4	.231
Pygospio	1.34	1.16	1.66	10.7	.001*	77	2.44	14	.017*
Scoloplos	1.48	0.70	3.16	1.52	.218	59	2.28	13	.022*
Tharyx	1.06	0.55	4.67	0.02	.899	63	1.79	2	.426

Note

For the frequency of occurrence approach the odds ratio for positive detection and its confidence interval were calculated, as well as Wald‐*χ* ^2^ and the area under the predictability curve. For the relative read abundance approach, slope of the linear regression, coefficients of determination (*R* ^2^) and *p*‐value are given.

* *p*< .05.

**FIGURE 5:**
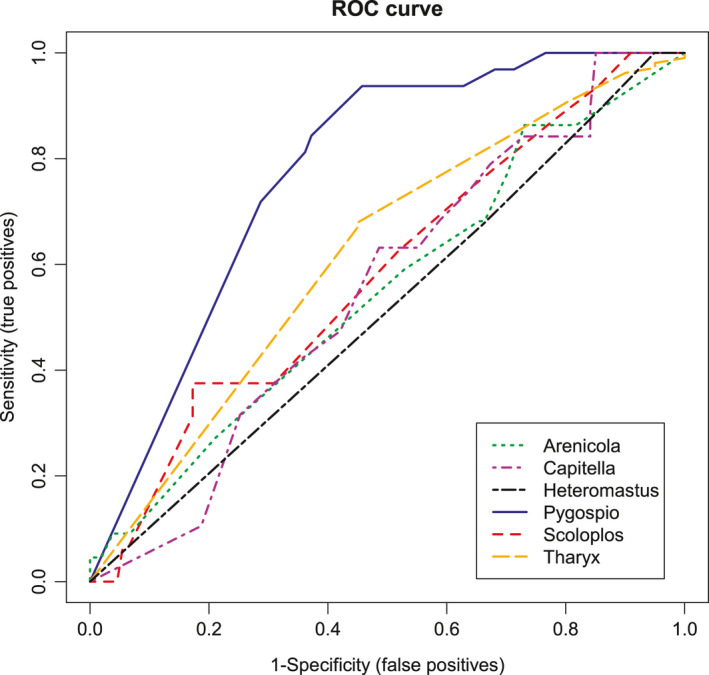
Receiver operating characteristics (ROC) curve as derived from the logistic regression for six annelid taxa. The ROC curve shows the sensitivity on the y‐axis, calculated as the true positive predictions by the logistic model and the specificity, calculated as the false positive predictions, on the x‐axis

The sensitivity and specificity for each taxon as based on the logistic regression model is shown in Figure 5 as a ROC curve. The degrees of measure for the predictive ability of the model were calculated based on these plots as the area under curve percentage (AUC, Table 2). The logistic model for *Pygsopio* was able to predict the presence of this taxon in the molecular approach at a 77% rate based on the abundance data in the morphological approach. *Scoloplos* had a predictability of roughly 60%, with a *χ* ^2^ of 1.52; however, for all other species, predictability was lower. The lowest predictability was found for *Heteromastus* and equalled 52%, which is close to a random guess.

**FIGURE 6:**
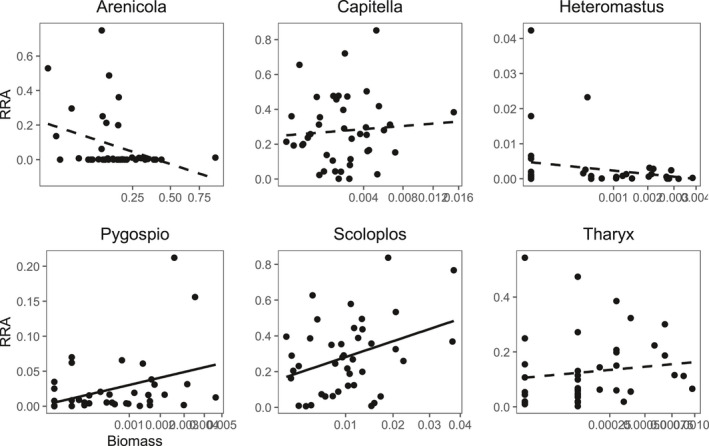
Relative read abundance approach relationships for six annelid taxa. The square‐root of biomass estimates as derived from the morphological method are shown on the x‐axis and the log_10_ of the relative read abundance of the same sample in the molecular data set is shown on the y‐axis

The relative read abundance as derived from the molecular method was modelled in a linear regression against the biomass estimates from the morphological method for six annelid taxa (Figure 6, Table 2). The steepest positive slope was found for *Pygospio*, followed by *Scoloplos* and *Tharyx*. The slope found for *Pygospio and Scoloplos* were significant (respectively, *F*_1,40_ = 6.25, *p* = .017, *R* ^2^ = .14 and *F*_1,40_ = 5.69, *p* = .022, *R* ^2^ = .13). *Capitella* and *Heteromastus* both showed no relationship between relative read abundance and biomass, while *Arenicola* displayed a (nonsignificant) negative slope.

The frequency of occurrence and the relative read abundance approaches both showed roughly the same results (Table 2). A strong positive relationship was obtained for *Pygospio* for both approaches. The relative read abundance approach also found a positive relationship for *Scoloplos*. Both approaches showed negative relationships for *Arenicola*.

## 4 DISCUSSION

The present study offers a insight in the quantitative abilities of DNA metabarcoding methods. We compared quantitative measurements for abundance and biomass for benthic macrofauna in the intertidal Dutch Wadden Sea for DNA metabarcoding approaches with traditional morphological approaches. Although some widely dispersed annelid taxa showed positive relationships between the outcome of both methods, most taxa did not show such a relationship.

### 4.1 Species detection

The combined use of a morphological as well as a molecular method to quantify the same benthic community allowed us to examine whether detection rates for benthic macrofauna taxa are comparable between the two methods. Although the overlap between species found with both methods is high, 23 and 35 taxa, respectively were found from which 22 taxa in both methods, the detection rates between methods within a taxon showed differences. The detection rates for most arthropod and mollusc taxa showed deviating results between the two methods, but the detection rates for the annelid taxa were comparable. The detection rate of arthropod taxa was higher in the morphological compared to the molecular method while the opposite was true for most molluscs. Additionally, several mollusc taxa (e.g., *Cerastoderma*, *Magallana and Mytilus*) detected by the molecular method were never detected by the morphological method. Explanations for the striking differences in output between the two methods for the different phyla could potentially be sought both in a methodological and an ecological context.

Methodological issues within the molecular method, which can hamper the assessment of a community, have been described extensively (e.g., Alberdi et al., 2018; Elbrecht & Leese, 2015; Kelly et al., 2019; Lanzén et al., 2017 and Piñol et al., 2019). Two key steps necessary to avoid technical biases emerge from these earlier studies: the use of PCR‐replicates and the use of a mock community. Alberdi et al. (2018) showed that considerable diversity differences exist between PCR replicates for the same sample, possibly due to PCR stochasticity and/or the accumulation of PCR errors. In the present study, three PCR replicates per sample were included to minimize these biases as suggested in Grey et al. (2018). Also, a mock community, an artificial community with known species was included to be able to identify biases induced in the metabarcoding process. This could be, for instance, biases induced during the sequencing process, during PCR due to a mismatch between a (group of) species and the universal primer, as well as biases induced in the bioinformatics pipeline (Leray & Knowlton, 2016). In our study, all species added to the mock community were recovered and the analysis of read numbers showed no indication of a bias in the detection rate of taxa from different phyla, as differences in read numbers were unrelated to phylum level. Hence, we assume that biases induced in the laboratory procedures are negligible and do not cause the discrepancy in detection rates for the molluscs and arthropods between both methods.

Another explanation can be sought in the ecological and biological features of eDNA and the host species. The eDNA in an environmental sample such as marine sediments is a mixture of DNA molecules which can include traces of organisms (e.g., air, faeces, mucus) rather than the organism itself (Taberlet, Coissac, et al., 2012). The amount of eDNA in the environment is a result of the biology (physical appearance, ontogenetic state, physiology) and ecology (seasonal and spatial patterns) of the taxa from which it originates (Harrison et al., 2019). In our data sets, we found a higher detection rate of mollusc with the molecular approach compared to the morphological approach. If we break down the detection rates of *Cerastoderma*, *Magallana* and *Mya* per sampling event, a potential seasonal pattern can be seen in which the peaks in detection rate coincided with known spawning periods of these taxa (Figure S1, Philippart et al., 2014). Second, the taxa *Mytilus* and *Magallana* are known to live in high densities in intertidal beds (Folmer et al., 2017) that were situated approximately 500 m from the sampled area during the study period. Even though specimens of these taxa were not found using the morphological approach, eDNA could easily have spread and be trapped within the sampled area due to tidal movements. The low detection rate of arthropods in the molecular samples compared to the morphological samples may also be explained by biological factors. Arthropods are characterized by an exterior skeleton made of chitin. This skeleton might inhibit eDNA exchange with the environment. Also, in contrast to mollusc taxa, the arthropod taxa found in this study rely on internal fertilization, which minimizes the extra production of eDNA due to spawning. Biases induced due to ecological characteristics of eDNA and its host species have been described scarcely but deserve a great deal of attention in the future (Stewart, 2019).

### 4.2 Quantification

The second aim of this study was to test the reliability of the frequency of occurrence and the relative read abundance approaches for quantifying abundance and biomass of marine benthic taxa with molecular methods. For this, we tested the underlying assumptions that a higher abundance or biomass in the morphological data set leads to a higher detection rate or relative read abundance in the molecular data set for the frequency of occurrence and relative read abundance approach, respectively (Deagle et al., 2019). We only found a positive relationship for *Pygospio* using the frequency of occurrence approach and for *Pygospio* and *Scoloplos* for the relative read abundance approach and not for any of the other species. Comparable results have been found by Bijleveld et al., (2018) in which they showed that occurrence data from a morphological data set on macrozoobenthic taxa in the intertidal Wadden Sea did not predict abundancies for these taxa. They discussed that the predictions in that study were highly influenced by dispersal and aggregation patterns and more reliable predictions could be made for taxa with higher dispersal rates. The same bias might also play a role in the present study. Adult *arenicola*, which has holobenthic development, has relatively low dispersal capacity, resulting in an aggregated distribution pattern (Günther, 1992). These *Arenicola* showed negative relationships in the frequency of occurrence approach, whereas *Pygospio*, which is distributed more evenly across mud flats (Gudmundsson, 1985), showed a strong positive relationship. This may be interpreted to indicate that species with an even spatial distribution also distribute their DNA evenly over the environment, while patchily distributed species correspondingly also have patchily distributed DNA, despite, for example, tidal water movements. DNA‐based frequency of occurrence of the more evenly distributed species would then typically not only be higher overall but also correlate better with morphology‐based quantifications than would be the case for patchily distributed taxa.

Compared to the frequency of occurrence approach, the relative read abundance is thought to be more influenced by methodological issues than by ecological issues (Deagle et al., 2019; Piñol et al., 2019). We hypothesized that the relative read abundance approach would be more influenced by the compositional nature of the molecular data set as relative abundances within an eDNA metabarcoding data set are negatively correlated, that is, an increased abundance of one species leads to a lower abundance for the other due to limited sampling depth (Gloor et al., 2017). However, the frequency of occurrence and relative read abundance gave comparable results in this study, which is a significant positive relationship for *Pygospio*. Moreover, relative read abundance approach also showed a significant positive relationship for *Scoloplos* between the relative read abundance and biomass.

The comparable results between the frequency of occurrence and relative read abundance methods might imply that both methods were subject to the same factors or biases in this study. We discussed that the ecological and biological features might be an important factor in this. The sampling strategy used in this study possibly induced biases in the quantitative measurements as well. Smaller species and species which are more evenly distributed such as *Pygospio* and *Scoloplos*, showed positive relationship for both approaches whereas bigger and more aggregated species such as mollusc species and *Arenicola* showed no relationship. For this study, a molecular sampling method was chosen in which only small sediment samples were collected from which DNA was extracted directly. Specimens of the smaller taxa might be physically present in the sample while larger metazoan taxa are sampled via their eDNA traces (Taberlet, Prud’homme, et al., 2012). Larger macrofauna species might have shown better quantitative relationship when bulk samples would have been used (Klunder et al., 2019). However, we chose the current method as it applies fewer treatments to the samples, which makes the method less susceptible to contamination (Aylagas et al., 2016; Elbrecht et al., 2017) as well as faster and easier to perform. In accordance with this, Elbrecht et al. (2017) found that read abundance of unsorted samples were dominated by taxa containing higher biomass within the sample and size sorting could help in preventing this bias. However, they also discuss size sorting should only be used when highly necessary to prevent cross‐contamination between the samples.

### 4.3 Future outlook

Metabarcoding methods have undergone considerable developments in the past decade and the methodology is still advancing rapidly. These advancements and new insights possibly harbour solutions to the current challenges and limitations in quantifying species abundancies from eDNA studies. It is outside the scope of this study to discuss them all, but we would like to highlight some. First, in this study we tested the conventional frequency of occurrence and relative read abundance approaches to quantify macrobenthic abundancies from eDNA studies. We showed that both approaches are limited in their quantitative abilities towards this community. In recent years, approaches have been developed that better incorporate the compositional conditions of eDNA data for microbial communities (Gloor et al., 2017) which are potential useful for all eDNA data (Quinn et al., 2019). Second, in this study it became clear that ecological and biological factors are important factors which can bias species detection and quantification. Ecological factors driving the distribution of eDNA have been described sparsely in the past and only gained scientific interest recently (Stewart, 2019). Moving forward, we suggest further knowledge about the factors driving eDNA distribution in the environment for different taxa and how this coincides with the sampling strategy of choice should be gained.

## CONFLICT OF INTEREST

The authors declare no conflict of interest.

## AUTHOR CONTRIBUTIONS

Lise Klunder, Allert I. Bijleveld, Henk W. van der Veer and Pieternella C. Luttikhuizen conceived the study; Lise Klunder, Loran Kleine Schaars and Pieternella C. Luttikhuizen performed the laboratory and fieldwork; Lise Klunder analysed the data and wrote the first draft of the manuscript; Lise Klunder, Allert I. Bijleveld, Pieternella C. Luttikhuizen, Henk W. van der Veer, Loran Kleine Schaars and Judith D.L. van Bleijswijk edited the manuscript.

## Supporting information

Appendix S1

## Data Availability

Raw Illumina sequences and corresponding metadata have been deposited in the European Nucleotide Archive; accession number PREJB46793. Sanger sequences from our internal reference library can be accessed through Genbank, accession nos. MZ709983–MZ710042.
