# Supplementary material for: Quantification of marine benthic communities with metabarcoding
Source: Mol Ecol Resour. 2021 Nov 1;22(3):1043–54. doi: 10.1111/1755-0998.13536 (PMC9298412; doi:10.1111/1755-0998.13536)
Supplement: Supplementary file 1 — Appendix S1 [file MEN-22-1043-s001.docx]

**Supplementary files**


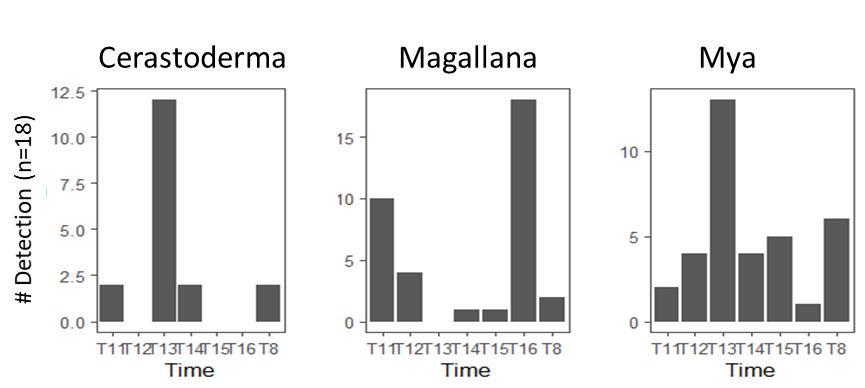


**Supp figure 1**: number of detections of Cerastoderma, Magallana and Mya within the molecular approach per sampling event (n=18). Time codes correspond to: T11 – November, T12 – March, T13/T14 – May, T15/T16 - June

**Supp Table 1: Mock community**

Mean read abundance for taxon found in the 4, similar, mock-communities which were added as positive controls. Taxa in bold are taxon groups which were added to the mock-community

| **Taxon** | Phylum | Mean reads (n=4) |
| --- | --- | --- |
| Carcinus | Arthropoda | 0.25 |
| Copepoda | Arthropoda | 0.25 |
| Mammalia | **-** | 0.25 |
| Phyllodoce | Annelida | 0.5 |
| **Melita** | **Arthropoda** | **1.5** |
| Alitta | Annelida | 1.75 |
| **Spionida_sp** | **Annelida** | **4.25** |
| Cerastoderma | Mollusca | 13.75 |
| **Scoloplos** | **Annelida** | **18.5** |
| Pygospio | Annelida | 21.75 |
| Nephtyidae_sp | Annelida | 29 |
| **Limecola** | **Mollusca** | **76.75** |
| **Pyllodocidae_sp** | **Annelida** | **86.25** |
| **Nemertii** | **Annelida** | **110** |
| **Hediste** | **Annelida** | **180.5** |
| **Lanice** | **Annelida** | **301.75** |
| **Crustacea_sp** | **Arthropoda** | **381.25** |
| **Marenzelleria** | **Annelida** | **552.25** |
| **Bivalvia_sp** | **Mollusca** | **836.5** |
| **Urothoe** | **Arthropoda** | **1104.75** |
| **Arenicola** | **Annelida** | **1162** |
| **Mya** | **Mollusca** | **1842** |
| **Metazoan_sp** |  | **1932.25** |
| **Crepidula** | **Mollusca** | **2199.5** |
| **Bathyporeia** | **Arthropoda** | **2293.75** |
| **Polychaeta_sp** | **Annelida** | **2532.75** |
| **Eteone** | **Annelida** | **4892.5** |
| **Ensis** | **Mollusca** | **5591** |
| **Gammarus** | **Arthropoda** | **6619.25** |
| **Bylgides** | **Annelida** | **7221.25** |
| **Mytilus** | **Mollusca** | **15850** |
| **Capitella** | **Annelida** | **43495.5** |

**Supp Table 2:** Oligo-nucleotide combinations tested for COI and 18S gene. Primer was tested in vitro, the score shows the percentage of positive outcomes of the diagnostic-PCR. Each primer was first tested on a subset of the reference collection from Supp. Table 2. Those with a 100% score were subsequently tested on the complete reference set.

| Reference | Gene | Length | Forward | Reverse | Score |
| --- | --- | --- | --- | --- | --- |
| Folmer et al., 1994 | COI | 658bp | LCO1490 | HCO2198 | 83 % |
| Lobo et al., 2013 | COI | 658bp | LoboF1 | LoboR1 | 50 % |
| Leray et al., 2013 | COI | 313bp | mlCOIintF | jgHCO2198 | 68 % |
| Hadziavdic et al., 2014 | 18S – V4/5 | 630bp | F-566 | R-1200 | 100 % |
| Stoeck et al., 2010 | 18S – V4 | 375bp | TAReuk454FWD1 | TAReuk454Rev3 | 83 % |
| Sinniger et al., 2016 | 18S – V1/2 | 450bp | SSU_F04 | SSU_R22mod | 100 % |

**Supp Table 3:** List of macrofaunal benthic species from the Dutch Wadden Sea in the morphological reference collection

| **Phylum** | **Species** |
| --- | --- |
| Annelida | Scoloplos armiger |
|  | Lanice conchilega |
|  | Nephtys hombergii |
|  | Arenicola marina |
|  | Marenzelleria viridis |
|  | Heteromastus filiformis |
|  | Bylgides sarsi |
|  | Hediste diversicolor |
|  | Phyllodoce muscosa |
|  | Tharyx sp. |
|  | Pygospio elegans |
|  | Captitella capitata |
|  | Allita virens |
|  | Allita succinea |
|  | Eteone longa |
|  | Glycera tridactyla |
|  | Oligochaeta |
|  |  |
| Arthropoda | Bathyporeia sarsi |
|  | Corophium arenarium |
|  | Corophium volutator |
|  | Urothoe poseidonis |
|  | Melita palmata |
|  | Gammarus locusta |
|  | Jaera albifrons |
|  |  |
| Mollusca | Cerastoderma edule |
|  | Limecola balthica |
|  | Mytilus edulis |
|  | Peringia ulvae |
|  | Retusa obtuse |
|  | Abra tenuis |
|  | Ensis leei |
|  | Mya arenaria |
|  | Crepidula fornicata |
|  | Kurtiella bidentate |
|  |  |
| Nemertea | Nemertea |
